# The Effect of Critical Shoulder Angle on Clinical Scores and Retear Risk After Rotator Cuff Tendon Repair at Short-term Follow Up

**DOI:** 10.1038/s41598-019-48644-w

**Published:** 2019-08-23

**Authors:** Tahsin Gürpınar, Barış Polat, Engin Çarkçı, Murat Eren, Ayşe Esin Polat, Yusuf Öztürkmen

**Affiliations:** 1 Istanbul Training and Resarch Hospital, Department of Orthopaedics and Traumatology, Istanbul, Turkey; 2 University of Kyrenia, Department of Orthopaedic and Traumatology, Kyrenia, Turkish Republic of North, Cyprus; 3 Dr. Akçiçek State Hospital, Department of Orthopedics and Traumatology, Kyrenia, Turkish Republic of North, Cyprus

**Keywords:** Bone, Outcomes research, Risk factors

## Abstract

The authors aimed to investigate whether standard acromioplasty can reduce critical shoulder angle (CSA) effectively and to investigate the effects of postoperative CSA on the clinical outcomes and retear rates. Patients are divided in to three groups: group 1 (24 patients): CSA under 35° before surgery, group 2 (25 patients): CSA over 35° before surgery and under 35° after surgery and group 3 (17 patients): CSA over 35° before and after surgery. Standard acromioplasty was performed if CSA is over 35 and no acromioplasty was performed if the CSA is already under 35. Preoperative and postoperative CSAs, UCLA, Constant-Murley clinical score and visual analog scale (VAS) pain score were measured. The size of the rotator cuff tear was classified by the Patte classification in preoperative MRI and the quality of the repair was evaluated as retear if discontinuity detected in the postoperative first year MRI. There were 31 female and 35 male patients with a mean age of 59.3 ± 4.5 years (range, 48–68) at the time of surgery. The mean CSA is reduced from 37.8° ± 1.4 to 34.9° ± 1.2 (p < 0.001) significantly for patients who underwent acromioplasty. In 25 (59.5%) of the 42 patients, the CSA was reduced to under 35°, whereas in the other 17 (40.5%) patients, it remained over 35°. The mean Constant and UCLA score was 46.4 ± 6.6; 18.5 ± 1.6 preoperatively and 82.4 ± 6.2; 31.1 ± 1.9 postoperatively respectively (p < 0,001). The mean VAS decreased from 4.94 ± 1.09 to 0.79 ± 0.71 (p < 0.001). No Clinical difference was seen between patients in which CSA could be reduced under 35° or not in terms of Constant-Murley score, UCLA and VAS score. Retear was observed in 2 (8.3%) patients in group 1, in 4 (16%) patients in group 2 and in 3 patients (17.6%) in group 3. There was not any significant difference between the patients who had retear or not in terms of neither the CSA values nor the change of CSA after the surgery. Standard acromioplasty, which consists of an anterolateral acromial resection, can reduce CSA by approximately 3°. This is not always sufficient to decrease the CSAs to the favorable range of 30°–35°. In addition, its effect on clinical outcomes does not seem to be noteworthy.

## Introduction

Rotator cuff tears (RCTs) are one of the most common causes of shoulder pain; however, the reason of tear is not fully understood. It is commonly accepted that there is not a single reason; instead, both intrinsic and extrinsic factors contribute to degeneration and ultimately cause tears. Among the extrinsic factors, abnormal acromial morphology and space narrowing under the acromion have been considered as an important cause, based on the findings of Neer’s studies on acromial morphology and subacromial impingement^1^. The current literature suggests that other scapular morphologies for instance, the anterolateral extension of the acromion and an increase in glenoid inclination can lead to rotator cuff tears^2^. The critical shoulder angle (CSA) combines these two parameters and it is described as the angle between the glenoid surface and a line connecting the inferior rim of the glenoid and the lateral tip of the acromion^3^.

Lateral acromion (higher CSA) creates a more-lateral deltoid origin and results in greater shear and lesser compressive vector of the deltoid on the glenohumeral joint^4^. In a mechanical model, Gerber *et al*. found that differences in CSA lead to considerable differences in joint forces^4^. They found that increased CSA is associated with decreased compressive and increased shear forces. They stated that an additional 35% of supraspinatus muscle force is needed to reach the stability equal to the normal shoulder anatomy when the CSA is increased to 38°. In an observational study, Moor *et al*. found CSA, between 30° and 35° on patients without neither osteoarthritis nor rotator cuff tear^3^. However, they indicated that angles >35° are associated with a high prevalence of RCTs, whereas shoulders with a CSA of <30° are more likely to be osteo-arthritic.

Katthagen *et al*. showed that acromioplasty is capable of reducing the CSA in a cadaver study^5^. However, the effects of surgical decreasing of the CSA under 35° are still unclear and there arevery few clinical studies in the literature on the effect of acromioplasty on patients with high CSAs.

Our hypothesis was that, CSA can be altered with acromioplasty therefore altering CSA may affect clinical outcomes and retear rates. Therefore, we aimed to investigate how CSA can be altered with a standard acromioplasty in patients with high CSA and to observe the effects of postoperative CSA on the clinical results.

## Materials and Methods

We obtained ethical approval for this study from the Institutional Review Board of Istanbul Training and ResearchHospital, Turkey (Reference No. 1044; July 21, 2017). All experiments were performed in accordance with and following the Declaration of Helsinki Principles. All methods were performed in accordance with the relevant guideline and regulations. All patients gave their written informed consent for prior to shoulder arthroscopy and the publication of their individual data. The surgical decision was made on the combination of patient’s clinical symptoms, shoulder X-rays and MRI results. Patients with full-thickness rotator cuff tear who did not respond to conservative treatment which included anti-inflammatory drugs, subacromial injections and physical therapy process for 6 months were appointed for arthroscopic rotator cuff repair. Exclusion criteria for the study were traumatic cuff tears, previous shoulder surgery, rheumatologic diseases, patients with significant degenerative arthritis in the preoperative X-rays, operative findings including subscapularis tendon tear or massive rotator cuff tears extending into the teres minor or coexisting labral tear and grade 3–4 cartilage damage not seen previously in the preoperative x-rays. Finally, a total of 64 patients met the inclusion criteria. Patients are divided into three groups: group 1: CSA under 35° before surgery, group 2: CSA over 35° before surgery and under 35° after surgery and group 3: CSA over 35° before and after surgery. Standard acromioplasty was performed if CSA is over 35 and no acromioplasty was performed if the CSA is already under 35°. Preoperative and postoperative CSAs, clinical scores and retear rates were measured and compared statistically. All data is collected and recorded on a workstation for further analysis.

### Surgical technique

**Figure 1:**
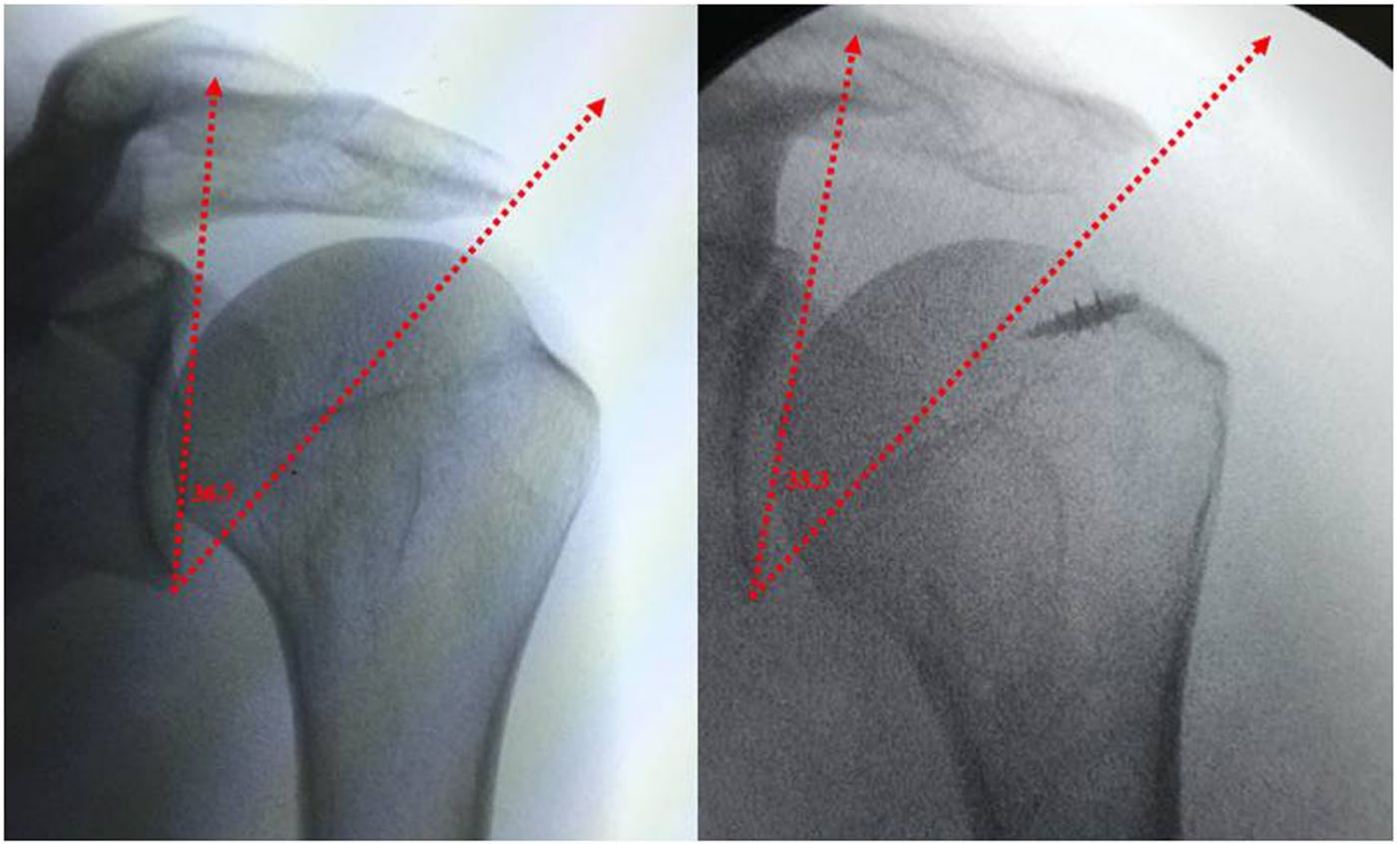
Preoperative and postoperative true AP shoulder view with fluoroscopy. CSAs value measured each view.

All of the operations were performed by the first surgeon in the bench chair position. The body section of the table was raised to 45°. The preoperative and postoperative CSAs of each patient were determined with fluoroscopy using a mobile C-arm. We preferred C-arm instead of X-ray because images can be taken under control of operating room staff, operation table and C-arm angle are adjusted to standardize images. The C-arm was positioned a 45° to the ground and 30° to the patient in order to superpose the anterior and posterior glenoid rim to obtain a true anteroposterior (AP) shoulder view (Fig. 1). All surgeries started with the establishment of a standard posterior portal and diagnostic arthroscopy. Anterior and lateral portals were performed regarding the tear location and size. After bursectomy and partial release of the coracoacromial ligament, lateral acromion was minimally resected (up to 5 mm) until reaching the deltoid attachments with an arthroscopic burr if preoperative CSA is over 35. Additionally, the anterolateral acromion was resected until the acromion was flattened. The acromioplasty is performed through the lateral portal while posterior portal was used for visualization. The amount of anterolateral resection differed from patient to patient, depending on the anatomy of the acromion. In all cases we preserved the deltoid attachment of the acromion despite the acromioplasty. After the acromioplasty, double row suture bridge rotator cuff repair was performed depending on the tear size and location. Immediately after the surgery, the true AP shoulder view was obtained by using the C-arm, as described above.

Postoperatively, each patient was prescribed a shoulder-immobilizing sling with an abduction pillow to maintain the shoulder at 30° abduction. Patients began gentle passive forward flexion and rotation on the first follow up at two weeks postoperatively. Patients were allowed to start active assistive range of motion (ROM) and stick exercises in 6 weeks and strengthening exercises began 10–12 weeks after surgery.

### Radiographic analysis

All CSA measurements were performed independently by 2 orthopaedic surgeon in order to assess the inter-rater agreement after surgical intervention. The decision of acromioplasty was given regarding the preoperative AP X-rays. Although the surgeon was informed whether the CSA was over 35 or not, the exact number of CSA was not given to avoid surgical bias. Inter-observer reproducibility was evaluated by comparing the numbers obtained by these two investigators and intra-observer reproducibility by having investigators repeat the measurements after three weeks. The CSA was measured as the angle subtended by a line parallel to the glenoid and a line through the inferior edge of the glenoid and the lateral edge of the acromion. The preoperative and postoperative CSA values were measured and compared statistically.

**Figure 2:**
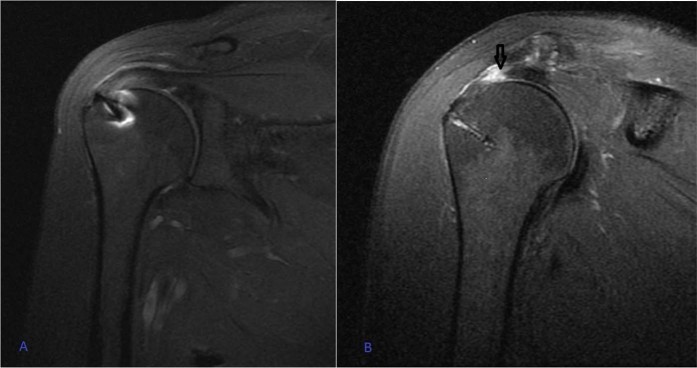
(**A**) Intact repair at the first year shoulder coronal MRI sequence in a group 2 patient. (**B**) Retear at the first year shoulder coronal MRI sequence in a group 3 patient. Black arrow shows Sugaya type 5 retear.

The MRI of shoulder were obtained preoperatively and 1 year postoperatively by 1.5-T MRI (Philips Medical System, Best, Netherlands). Both senior orthopedic surgeon (T.G. and B.P.) evaluated the preoperative and 1 year postoperative MRI scans. The size of the rotator cuff tear was classified by the Patte classification^6^ before time of surgery. In our study, 17 (40.5%) of the patients had stage 1 full thickness rotator cuff tears, 25 patients (59.5%) had stage 2 full thickness rotator cuff tears. The quality of the repair was evaluated as retear if identified as Sugaya type 4 and 5 (presence of a minor or major discontinuity) in the postoperative first year MRI^7^(Fig. 2).

### Clinical assessment

Age at the time of surgery, sex, side, tear size and retraction on MRI were recorded preoperatively for all enrolled patients. All patients were assessed clinically the day before the surgery. Postoperative evaluations were performed at 2 weeks, 4 weeks, 6 weeks, 3 months, 6 months, 9 months, and 12 months after the operation and at the last follow-up. For the clinical assessment, preoperative and postoperative Constant-Murley score and UCLA score were used. Subjective pain score was measured with the visual analogue scale (VAS) before and after the operation.

### Statistical methods

SPSS 15.0 for Windows program was used for statistical analysis. Descriptive statistics; number and percentage for categorical variables, mean, standard deviation, minimum, maximum, median for numerical variables were given. Paired t test was used when the two groups of dependent variables were normally distributed, otherwise the Wilcoxon test was used. Paired t test was used when the two groups of independent variables were normally distributed, otherwise the Wilcoxon test was used. Rates in dependent groups were compared with Mc Nemar test. One Way ANOVA test was used when the independent two groups were compared with the normal distribution condition of the numerical variables, and the Kruskal Wallis test was used when the normal distribution condition was not satisfied. In nonparametric test, subgroup analysis was performed with Mann Whitney U test and interpreted with Bonferroni correction. Statistical significance level of alpha was accepted as p < 0.05. Interobserver and intraobserver agreement was assessed using the ‘κ’ statistical test. A kappa value between 0.8 and 1 was considered perfect agreement.

## Results

There were 31 female and 35 male patients with a mean age of 59.3 ± 4.5years (range, 48–68) at the time of surgery. In terms of the extremities of the patients, 39 (59.1%) were affected in the right shoulder, whereas 27 (40.9%) were affected in the left shoulder. 42 (63.6%) of the patients underwent acromioplasty in addition to rotator cuff repair while 24 (36.4%) of them did not undergo acromioplasty (Table 1). The mean preoperative CSA was 37.8° ± 1.4 and the mean postoperative CSA was 34.9° ± 1.2 for patients who underwent acromioplasty. Therefore, the mean CSA of the patients was reduced significantly (p < 0.001) by a mean of 2.94° ± 0.41 after the acromioplasty described above (Table 2). In 25 of 42 (59.5%) patients, the CSAs could be reduced under 35° with this surgical method. The preop CSA and mean CSA change of patients whom postop CSA over 35° were statistically higher than postop CSA < 35 patients (p < 0.001 p = 0.033) (Table 3). Intra-observer reproducibility was 97.5% and inter-observer reproducibility was 94.7% for measurements of CSA.

**Table 1:** Patients demographics.

Age Mean ± SD (min-max)		59.3 ± 4.7 (48–68)
Gender n (%)	Male	35 (53.0)
	Female	31 (47.0)
Side n (%)	Right	39 (59.1)
	Left	27 (40.9)
Preop CSA		36.2 ± 2.5 (31.6–40.4)
Acromioplasty n (%)	Performed	42 (63.6)
	Not performed	24 (36.4)
Follow-up time (month)Mean ± SD (min-max)		17.5 ± 3.8 (12–26)

**Table 2:** The preoperative and postoperative CSA values of patients whom CSA value above 35 preoperatively.

Acromioplasty		Mean ± SD	Min-Max	Median
CSA	Preop	37.8 ± 1.4	35.4–40.4	37.6
	Postop	34.9 ± 1.2	33–37.2	34.7
	Differences	2.94 ± 0.41	1.9–3.7	95% CI: 2.81–3.07
	p*	<**0.001**		

p* Paired t test (statistically significant p values are written in bold).

**Table 3:** Comparison of preop CSA and CSA changes between group 2 and group 3.

		Preop CSA				
		N	Mean ± SD	Min-Max	Median	p
Postop CSA	<35 (group 2)	25	36.9 ± 0.8	35.4–38.2	37.1	<**0**.**001**
	>35 (group 3)	17	39.2 ± 0.7	38.2–40.4	39.2	
		**CSA diffrence**				
		**N**	**Mean** ± **SD**	**Min-Max**	**Median**	**p**
Postop CSA	<35 (group 2)	25	2.83 ± 0.35	1.9–3.5	2.9	**0**.**033**
	>35 (group 3)	17	3.10 ± 0.44	1.9–3.7	3.2	

(statistically significant p values are written in bold).

The mean Constant score was 46.4 ± 6.6 (range, 32–59) preoperatively and 82.4 ± 6.2 (range, 68–94) postoperatively at the last follow-up (p < 0.001). The mean UCLA score was 18.5 ± 1.6 (range, 16–22) preoperatively and 31.1 ± 1.9 (range, 26–34) postoperatively at the last follow-up (p < 0.001). The patients experienced substantial pain relief after their rotator cuff repair. The mean pain score on the visual analog scale decreased from 4.94 ± 1.09 (range 3–7) to 0.79 ± 0.71 (range, 0–2) (Table 4). There was no statistically significant difference in the mean age, gender side ratio and follow-up period of the patients in group 1, group 2 or group 3 (Table 5). No statistical difference has seen between patients in which CSA could be reduced under 35° or not in terms of preoperative and postoperative constant-murley score, UCLA score and VAS score. Hovewer, postoperative constant score was slightly higher in patients whose CSA was below 35° preoperatively (Table 6).

**Table 4:** The preoperative and postoperative clinical scores

		Mean ± SD	Min-Max	Median
Constant	Preop	46.4 ± 6.6	32–59	46
	Postop	82.4 ± 6.2	68–94	83
	Difference	36.0 ± 8.8	95% CI: 33.8–38.2	
	P	<**0.001**		
UCLA	Preop	18.5 ± 1.6	16–22	18.5
	Postop	31.1 ± 1.9	26–34	31
	Difference	12.6 ± 2.4	95% CI: 12.0–13.2	
	P	<**0.001**		
VAS	Preop	4.94 ± 1.09	3–7	5
	Postop	0.79 ± 0.71	0–2	1
	Difference	4.15 ± 1.10	95% CI: 3.88–4.42	
	P	<**0.001**		

p* Paired t test p**Wilcoxon Test (statistically significant p values are written in bold).

**Table 5:** Demographic characteristics of patients in group 1, group 2 and group 3.

		Group 1	Group 2	Group 3	
Age Mean ± SD (min-max)		59.2 ± 5.1 (48–68)	60.7 ± 4.0 (52–67)	57.6 ± 4.6(49–64)	0.105^#^
Gender n (%)	Male	13 (54.2)	12 (48.0)	10 (58.8)	0.781
	Female	11 (45.8)	13 (52.0)	7 (41.2)	
Side n (%)	Right	13 (54.2)	18 (72.0)	8 (47.1)	0.225
	Left	11 (45.8)	7 (28.0)	9 (52.9)	
**Follow-up time (month)**					
Mean ± SD (min-max)		17.3 ± 3.5 (12–24)	17.7 ± 3.8 (12–25)	17.8 ± 4.4 (12–26)	0.892^#^

^#^One-Way ANOVA.

**Table 6:** Comparison of clinical scores in group 1 (preop CSA < 35), group 2 (postop CSA < 35) and group 3 (postop CSA > 35).

		Postop CSA						p
		Group 1		Group 2		Group 3		
		Mean ± SD	Median	Mean ± SD	Median	Mean ± SD	Median	
Constant	Preop	46.4 ± 7.4	46	46.8 ± 6.4	47	45.8 ± 6.2	46	0.899^#^
	Postop	84.8 ± 5.7	85	80.2 ± 5.9	81	82.2 ± 6.2	84	**0.035** ^**#**^
	p	<0.001**		<0.001*		<0.001*		
UCLA	Preop	18.6 ± 1.7	18,5	18.8 ± 1.7	19	18.1 ± 1.5	18	0.359^≠^
	Postop	31.6 ± 1.8	32	30.4 ± 2.0	30	31.5 ± 1.7	32	0.056^≠^
	p	<0.001*		<0.001*		<0.001**		
VAS	Preop	4.75 ± 1.22	4,5	5.12 ± 1.17	5	4.94 ± 0.75	5	0.430^≠^
	Postop	0.67 ± 0.70	1	0.76 ± 0.60	1	1.00 ± 0.87	1	0.400^≠^
	p	<0.001*		<0.001**		<0.001**		

^#^One-Way ANOVA^≠^Kruskal Wallis test p* Paired t test p**Wilcoxon Test (statistically significant p values are written in bold).

Retear was observed in 2 (8.3%) patients in group 1 (preop CSA < 35), in 4 (16%) patients in group 2 (preop CSA ≥ 35° and postop CSA < 35°) and in 3 patients (17.6%) in group 3 (preop and postop CSA ≥ 35°). There was not any significant difference between the patients who had retear or not in terms of neither the CSA values nor the change of CSA after the surgery (Table 7).

**Table 7:** Retear rates between groups.

		Group 1		Group 2		Group 3		p
		n	%	n	%	n	%	
Retear	present	2	8,3	4	16,0	3	17,6	0,730
	absent	22	91,7	21	84,0	14	82,4	

## Discussion

CSA is defined as the angle between the glenoid surface and a line connecting the inferior rim of the glenoid and the lateral tip of the acromion, that concerns both the glenoid inclination and acromion which were identified as the cause of pathogenesis of the rotator cuff tears^3^. Neer focused on the mechanical impinging role of the acromion on the rotator cuff and he called this condition impingement syndrome^8^. Many authors have focused on the supraspinatus outlet and suggested that a hooked or a curved acromion could reduce the subacromial space and therefore irritate the rotator cuff tendons^9,10^. Although many studies using cadaveric models or 3-D models have been published that have investigated the correlation between acromial shape and rotator cuff tears, significant controversy still exists and it remains unclear whether the morphologic changes in the acromion are a reason or a consequence of rotator cuff tear^11–13^. This new concept of the CSA takes into account not only acromial cover but also the inclination of the glenoid. In addition, the line connecting the inferior rim of the glenoid and the lateral tip of the acromion can be affected both from lateral extension of acromion (which can alter deltoid biomechanics) and inferior bone spurs (which may cause impingement) on supraspinatus outlet.

Recently, theories concerning the pathogenesis of tears have focused on the shoulder biomechanics^14^. It is known that stability of the shoulder joint is only minimally provided by osseous constraints and it mostly relies on passive structures and active rotatory muscle forces. Contraction of the deltoid during active abduction pulls the humeral shaft upward and presses the humeral head through the glenoid fossa. An imbalance in the interaction of the deltoid and antagonist forces may diminish concavity compression and furthermore, may cause to elevation of the humerus^15^. This superior elevation would be compensated by the rotator cuff in order to stabilize the humeral head within the glenoid fossa. Nyffeler *et al*. suggested that the lateral extension of the acromion alters the orientation of the deltoid fibres at their origin, thus altering the resultant deltoid muscle force vector^2^. Larger lateral extension of the acromion increases the ascending force, which may lead subacromial impingement and degenerative changes of the supraspinatus tendon^2^. Therefore, in theory acromioplasty can decrease this ascending force, reduce CSA and reduce the amount of forces on supraspinatus. However, in our study we could not find any relation between CSA and the clinical results. On the other hand, the forces on supraspinatus after acromioplasty is beyond the scoop of our study.

The other factor impacting CSA is the glenoid shape. Greater obliquity of the glenoid lead to an increase in the shear component of the forces created by the deltoid muscle^16^. Wong *et al*. investigated the role of glenoid inclination in a biomechanical study and they found that the force required to produce superior humeral head subluxation reduced significantly as the glenoid inclination angle increased^17^. Furthermore, Hughes *et al*. observed that the glenoid inclination was significantly superior in the shoulders with full-thickness rotator cuff tears in their cadaver study^16^. In a cadaver model Konrad *et al*. simulated a massive RCT and showed that decreased glenoid inclination significantly reduces superior humeral head translation at glenohumeral abduction^18^. However, glenoid inclination cannot be addressed with acromioplasty. Although CSA takes into account both acromion shape and glenoid version, acromioplasty can only change one of the two variables of CSA. Even if we can alter the CSA with acromioplasty, the expectation of biomechanical change originating from glenoid inclination would be unrealistic. This can be one of the reasons that we did not find any clinical difference between groups.

Although the effect of altering CSA on functional outcomes or on the retear rates of rotator cuff repair is not clear, the authors speculated that acromioplasty could potentially reduce a CSA greater than 35° to the range of 30° to 35°, which could lower the risk of retear after rotator cuff repair. Katthagen *et al*. showed that acromioplasty is capable of reducing the CSA in a cadaver study^5^. On the other hand, in the present study, it was not possible to reduce almost half of the CSAs under 35°. A more aggressive lateral acromion resections in patients with very high CSA could reduce their CSA to below 35°, however this could comprimise deltoid atachment. Inspite of this concern, recent studies stated that lateral acromial resection up to 6 mm showed no harm to deltoid attachment and function^5,19^. In our study, we have not seen any clinical and retear rate difference between groups in which CSA could be reduced under 35 or not. The technique used in this case was similar to the technique performed by Katthagen *et al*., although their native mean CSA (34.3) was already under 35°. Therefore, it cannot be assumed that it is always possible to reduce CSA under 35° with standard acromioplasty. Although the CSA was reduced by a mean of 2.9°, it was not sufficient for all of the cases and further lateral acromial resection was not performed in these cases due to concern for deltoid insertion. It may be speculated that these patients have wider acromion and therefore maybe more tolerant of lateral acromial resection but without a proved advantage we did not attempt to reduce CSA. In addition, we have not found any difference between group 1, group 2 or group 3 in terms of re-tear rates. Although the postop constant score was slightly higher in patients whose CSA was below 35° preoperatively, the difference was very small and under the minimal clinical importance score which was found as 10.4 previously^20^. Therefore, it is more likely that CSA has no effect on clinical results after rotator cuff repair.

Controversy still remains about the effect of CSA on clinical results. The only study supporting the influence of postoperative CSA was published by Gerber *et al*. in which they found inferior results with latissimus dorsi transfer on patients with large CSA^4^. On the other hand, some authors suggest that an increased CSA does not correlate with rotator cuff disease and does not affect the postoperative outcomes^21,22^. Kirsch *et al*. examined the relationship between the CSA and outcomes after rotator cuff repair but they were unable to find any effect of CSA on clinical results after arthroscopic full-thickness rotator cuff tears^22^. Likewise, Chalmers *et al*. investigated 326 radiographs and, they found mean CSA 34° ± 4° among patients with cuff tears and 32° ± 4° among control subjects. Although they found CSA 2.0° higher in patients with rotator cuff tears, they concluded that the difference was small and therefore, CSA is not related to rotator cuff disease^21^. In spite of this, Scheiderer *et al*. concluded that high CSA increased retear rate after arthroscopic tendon repair^23^. They evaluated 57 patients retrospectively and found that the mean CSA for the retear group (mean 37°) was significantly higher than that in the intact group (35°). Both Chalmers *et al*.^21^ and Scheiderer *et al*.^23^ found similar results but they disagreed on the clinical importance of 2° difference. In our study we could decrease the CSA approximately 3° with standard acromioplasty and the change of CSA was statistically significant. However statistical significance sometimes does not imply clinical importance and in our study this 3° of change did not effect clinical results. After mean 17.7 months we have not seen any difference either on clinical results or retear rates between patients whose CSA could be reduced under 35° or not. Patients clinical results were satisfactory in all of the groups. Therefore, we cannot advocate to reduce the CSA under 35° in all cases. However, there is still a lack of prospective randomized studies in the literature and further studies should focus on the effect of surgical altering of CSA on clinical results.

This study has some limitations. The acromioplasty described does not resect the same amount of bone in all cases, depending on the anatomy of the patient. All cases were performed by the same surgeon in the present study, but the term “standard acromioplasty” can vary from surgeon to surgeon; therefore, the effect of acromioplasty can change. In a limited number of cases, we could not find a significant relationship between the postoperative CSA with clinical outcome and retear rate. In addition, sample size in this study is not large enough to determine a statistical difference in retear ratios. More extensive studies are needed to determine this relationship. On the other hand, this is the first study to attempt to investigate the effect of acromioplasty on shoulders with CSAs over 35°.

## Conclusions

This study shows that standard acromioplasty, which consists of minimally lateral and anterolateral acromial resection, can reduce CSA by approximately 3°. This is not always sufficient to decrease the CSAs to the favorable range of 30°–35°. In addition, its effect on clinical outcomes does not seem to be noteworthy.

## Data Availability

The corresponding author had full access to all the data in the study and all authors shared final responsibility for the decision to submit for publication.
